# Association of obesity-related anthropometric indicators with chronic constipation and diarrhea among U.S. adults: a cross-sectional study

**DOI:** 10.3389/fnut.2025.1610214

**Published:** 2025-08-18

**Authors:** Yu Ning, Xiaoting Hu, Laifu Li, Yan Zhuang, Fei Dai

**Affiliations:** Department of Gastroenterology, The Second Affiliated Hospital of Xi'an Jiaotong University, Xi'an, China

**Keywords:** obesity, anthropometric indicators, constipation, diarrhea, NHANES

## Abstract

**Aim:**

Prior studies have linked obesity indicators to constipation/diarrhea, but multi-measure comparisons remain limited. We analyzed these associations in U.S. adults.

**Methods:**

This cross-sectional study utilized data from three cycles (2005–2010) of the National Health and Nutrition Examination Survey (NHANES). The final analysis included 13,105 participants after excluding those aged < 20 years or with missing data for any study variables. Bowel habits were categorized using the Bristol Stool Form Scale (BSFS). Multiple analytical approaches were employed: descriptive statistics, weighted multivariable logistic regression, weighted restricted cubic spline (RCS) analysis, subgroup analyses, and sensitivity analysis. We evaluated the diagnostic performance of various anthropometric indices—waist circumference (WC), body mass index (BMI), relative fat mass (RFM), body roundness index (BRI), weight-adjusted waist index (WWI), waist-to-height ratio (WHtR), and a body shape index (ABSI)—for chronic diarrhea and constipation using receiver operating characteristic (ROC) curve analysis and the area under the curve (AUC).

**Results:**

Weighted multivariable logistic regression revealed significant positive associations between seven obesity indicators and diarrhea (all *P* < 0.05), with the highest odds ratios (ORs) observed in the top quartiles for WWI (OR = 1.937, 95% CI = 1.516–2.474, *P* < 0.001) and RFM (OR = 1.870, 95% CI = 1.254–2.790, *P* = 0.003). Meanwhile, RFM, BRI, WC, BMI, and WHtR showed significant inverse associations with constipation (*P* < 0.05), with the lowest ORs observed for the top quartiles of BMI (OR = 0.530, 95% CI = 0.408–0.689, *P* < 0.001) and RFM (OR = 0.599, 95% CI = 0.409–0.879, *P* = 0.011). By contrast, the top ABSI quartile exhibited a positive association with constipation (OR = 1.262, 95% CI = 1.014–1.571, *P* = 0.038). ROC analysis indicated RFM as the most discriminative indicator for constipation (AUC = 0.577) and WWI for diarrhea (AUC = 0.614), respectively, among tested indices. RCS analysis demonstrated an inverse J-shaped relationship between RFM and constipation and a linear positive association between WWI and diarrhea. Subgroup analyses further validated the robust associations of RFM and WWI with intestinal disorders across strata of age, sex, race, smoking, drinking, sleep disturbances, diabetes, and depression. Sensitivity analyses yielded consistent results, supporting the stability of these findings.

**Conclusions:**

The seven indicators are useful indicators for assessing intestinal disorders in U.S. adults, with RFM and WWI demonstrating the highest discriminative ability for constipation and diarrhea, respectively.

## 1 Introduction

With economic development, dietary patterns and lifestyles have undergone significant changes, leading to an increased prevalence of gastrointestinal disorders. Chronic diarrhea is characterized by increased bowel frequency and loose stools persisting for more than 4 weeks, with a prevalence of approximately 11%−30% in the U.S. population, affecting about 6.6 % of adults (1–3). Chronic constipation is defined as fewer than three bowel movements per week, typically accompanied by hard or lumpy stools, incomplete evacuation, and bloating, and affects 9%−20% of U.S. adults (4, 5). Chronic constipation and diarrhea, as prevalent gastrointestinal disorders worldwide, pose significant challenges to public health. These disorders substantially impair patients' quality of life while imposing considerable financial strain on healthcare systems (6, 7).

Global obesity rates and associated diseases are steadily increasing, surpassing 2 billion overweight individuals worldwide (8). Recent epidemiological data (2021) reveal that approximately 21.4 million U.S. individuals aged 15–24 years and 172 million aged ≥25 years met diagnostic criteria for overweight or obesity. Obesity rates are escalating steadily, with projections indicating that 50% of U.S. adults will meet obesity criteria by 2030, while combined overweight/obesity prevalence may surpass 80% by mid-century (9, 10). Obesity raises the risk of cardiovascular diseases, diabetes, and various cancers, including colon, stomach, breast, and kidney cancer. It is also significantly associated with non-neoplastic gastrointestinal disorders. Excess adiposity elevates pro-inflammatory cytokines (TNF-α, IL-6) and leptin while suppressing adiponectin, driving chronic low-grade inflammation, intestinal barrier dysfunction (increased permeability), and gut microbiota dysbiosis; meanwhile, central obesity increases intra-abdominal pressure and gastric acid secretion while reducing lower esophageal sphincter pressure and length, further impairing esophageal motility (11, 12). Substantial evidence indicates an elevated susceptibility to chronic diarrhea among obese populations (12, 13). However, the association of obesity with constipation remains complex and inconsistent. Some studies show higher rates of constipation in adults with class II and III obesity, while others have shown that low BMI and reduced abdominal fat are associated with constipation risk and hard stools. Furthermore, some studies have found no significant association between obesity and functional constipation (14–17).

While BMI serves as a simple tool for assessing weight status, it fails to distinguish adipose tissue from muscle mass or characterize fat distribution. WC provides an alternative method to estimate visceral fat (18). However, the traditional use of BMI and WC may not adequately capture their associations with obesity-related diseases. In recent years, numerous cost-effective and innovative indices have emerged, enabling more precise and comprehensive multidimensional assessment of obesity. For instance, the RFM estimates body fat content and exhibits superior accuracy over BMI in predicting whole-body fat percentage in both male and female (19). The WWI independently reflects central obesity and excels in accurately assessing both central and visceral obesity (20). The BRI captures body roundness and fat distribution patterns, particularly central obesity, while the ABSI reflects abdominal fat distribution, especially visceral fat accumulation (21, 22).

The aim of this study was to investigate associations between multiple obesity-related indicators and both constipation and diarrhea, identify the strongest predictors for each condition among tested indices, and provide evidence to inform clinical prevention and treatment strategies.

## 2 Methods

### 2.1 Study design and participants

This is a cross-sectional analysis of publicly available data from three consecutive NHANES cycles (2005–2006, 2007–2008, and 2009–2010) that included bowel-health assessments. The NHANES is an ongoing epidemiological surveillance program conducted by the Centers for Disease Control and Prevention (CDC) to evaluate the health and nutritional status of the U.S. population (23). The survey was approved by the Ethics Review Board of the National Center for Health Statistics (NCHS), with all participants providing voluntary informed consent. Additional information is freely available on the NHANES website (https://www.cdc.gov/nchs/nhanes/index.htm).

**Figure 1:**
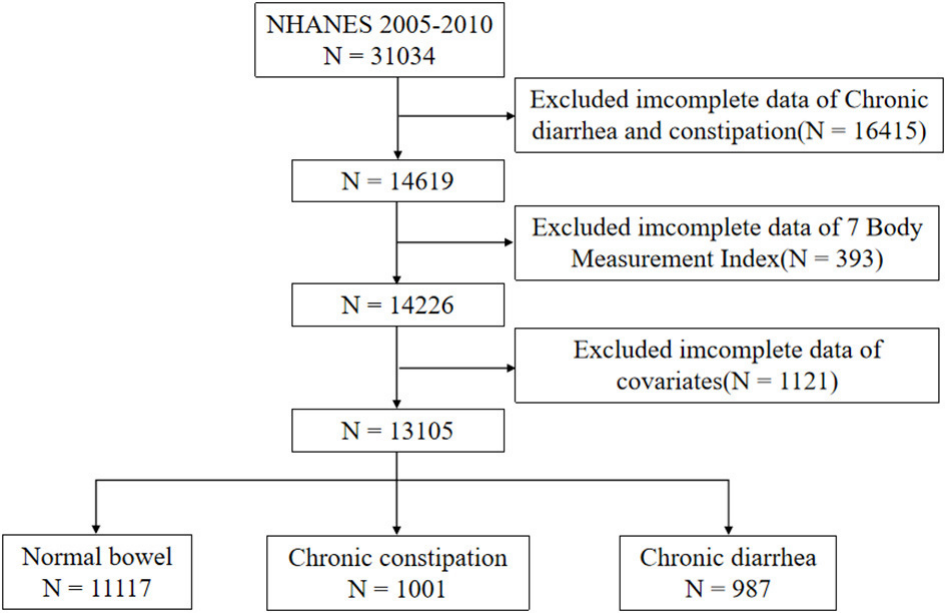
Flowchart of participant selection process.

The study engaged 31,034 participants. Exclusion criteria included the following: age under 20 years, missing bowel habits, missing information on the seven body measurements (WC, BMI, RFM, BRI, WWI, WHtR, ABSI), and missing information on covariates. We ended up with 13,105 eligible subjects. Figure 1 details the participant screening process.

### 2.2 Variables

#### 2.2.1 Bowel health questionnaire

The study employed the Bowel Health Questionnaire for bowel disorder classification. During assessments, participants referenced Bristol Stool Form Scale (BSFS) visual aid to describe stool types 1–7. Following established criteria, chronic constipation was characterized by predominantly BSFS Types 1 or 2 (hard/lumpy stools), while chronic diarrhea comprised Types 6 or 7 (mushy/watery stools). All other participants were categorized as having normal bowel habits (20, 24).

#### 2.2.2 Obesity-related anthropometric indicators

To examine adiposity-bowel habit associations, we analyzed directly measured anthropometric data from NHANES and incorporated seven obesity-related indicators: WC, BMI, RFM, BRI, WWI, WHtR, and ABSI. WC = horizontal circumference around the umbilicus (cm). BMI = weight (kg)/[height (m)]^2^. RFM = 64 – (20 × height (m) / WC (m)) + (12 × sex), where sex = 1 for female and 0 for male. BRI = 364.2 – 365.5 × √[1 – (WC/(2π))^2^/(0.5 × height)^2^]. WWI is calculated by dividing WC (cm) by the square root of weight (kg). WHtR = WC (cm)/height (cm). ABSI = WC (m)/[BMI^(2/3)^^*^ height (m)^(1/2)^]. All measurements were performed by certified NHANES staff using calibrated equipment. These indices were selected due to their practicality in assessment and general acknowledgment (19–22).

#### 2.2.3 Covariates

Potential confounders were adjusted as covariates. The demographic characteristics included: age (>20), sex, race (Mexican American, non-Hispanic White, non-Hispanic Black, other), marital status, and education level (below high school, high school, and above high school), and socioeconomic level [Low income: Poverty income ratio (PIR) < 1.3; Middle income: 1.3 ≤ PIR < 3.5; High income: PIR ≥ 3.5]. Furthermore, we incorporated various self-reported lifestyle factors and clinical measures, including alcohol consumption (drinker: ≥12 standard drinks/year; non-drinker: < 12 drinks/year), smoking (never smokers, current smokers, and former smokers), sleep disturbances, mental health [Not Depressed: Patient Health Questionnaire-9 (PHQ-9) score < 10; Depressed: PHQ-9 score ≥ 10], and diabetes mellitus.

### 2.3 Statistical analyses

To enhance the representativeness of our findings, we selected WTMEC2YR/3 as the analytic weights in accordance with NHANES analytical guidelines. Baseline characteristics of individuals with normal bowel function, chronic diarrhea, and chronic constipation were summarized using descriptive statistics, with continuous variables as mean ± SD and categorical variables as proportions. Three progressively adjusted weighted logistic regression models analyzed associations between seven adiposity indices and bowel disorders: Model 1 (crude), Model 2 (demographically adjusted for age, sex, race), and Model 3 (further adjusted for education level, socioeconomic level, smoking, drinking, sleep disturbances, depression, and diabetes). The predictive capacity was evaluated through ROC-AUC analysis, with RFM and WWI additionally assessed via weighted RCS regression for non-linear effects. Stratified analyses were conducted by demographic characteristics (sex; age < 60/≥60 years; race), lifestyle factors (drinking, smoking, sleep disturbances), and clinical status (depression, diabetes mellitus). For sensitivity analysis, after excluding participants with potential confounding factors (including pregnancy, colorectal cancer, or use of gastrointestinal drugs, psychotropic medications, or opioids), we performed weighted logistic regression and ROC curve analyses with additional adjustments for covariates including total fat intake, dietary fiber, protein, carbohydrates, total sugar, caffeine, total energy intake, and physical activity levels. Dietary data were collected using standardized 24-h dietary recall questionnaires, while physical activity was quantified by multiplying metabolic equivalent (MET) values by weekly exercise duration and categorized into three levels: low (< 600 MET-min/week), moderate (600–3,000 MET-min/week), and high (>3,000 MET-min/week). Statistical significance was defined as *P* < 0.05. Analyses were performed using R 4.4.2 with the *tableone, survey, pROC, rms, do, ggplot2 and ggpubr* packages.

## 3 Results

### 3.1 Baseline characteristics

The study enrolled 13,105 participants, comprising 11,117 with normal bowel function, 987 with chronic diarrhea, and 1,001 with chronic constipation. Chronic diarrhea and constipation patients averaged 49.81 ± 15.80 and 45.01 ± 17.46 years, respectively, with significant female predominance (58.36% and 71.4%, respectively). The study population was predominantly non-Hispanic White, accounting for 67.97% of the chronic diarrhea group and 65.83% of the chronic constipation group. Complete baseline characteristics are presented in Table 1. The weighted descriptive analysis revealed significant differences in diarrhea patients regarding sex, race, education level, marital status, socioeconomic level, drinking, smoking, sleep disturbances, mental health, diabetes, and seven obesity-related indicators (all *P* < 0.05). For constipation patients, the differences in sex, race, education level, marital status, socioeconomic level, drinking, smoking, mental health, BMI, WC, and RFM were statistically significant (all *P* < 0.05).

**Table 1:** Weight-adjusted population characteristics.

**Variable**	**Normal bowel *N* = 11,117**	**Constipation *N* = 1,001**	***P*-value**	**Diarrhea *N* = 987**	***P*-value**
**Age (years)**	46.24 ± 16.38	45.01 ± 17.46	0.0418	49.81 ± 15.80	<0.0001
**Sex (%)**			<0.0001		<0.0001
Male	50.88	28.60		41.64	
Female	49.12	71.40		58.36	
**Race (%)**			<0.0001		0.0058
Mexican American	7.62	9.08		9.75	
Non-Hispanic White	10.27	65.83		67.97	
Non-Hispanic Black	72.85	14.74		12.07	
Others	9.26	10.35		10.20	
**Education level (%)**			<0.0001		<0.0001
Below high school	16.67	22.07		26.40	
High school	23.81	28.51		25.14	
Above high school	59.52	49.42		48.46	
**Marital status (%)**			0.0087		0.0023
Divorced/separated	12.51	14.41		14.23	
Married or living with a partner	66.11	59.79		65.31	
Never married	16.41	18.48		13.13	
Widowed	4.98	7.31		7.33	
**Socioeconomic level (%)**			<0.0001		<0.0001
Low income	17.94	25.07		24.83	
Middle income	35.62	39.80		35.07	
High income	46.44	35.13		40.10	
**Smoking (%)**			0.0006		0.0041
Current smoker	22.27	20.21		27.10	
Former smoker	25.11	19.97		26.98	
Never smoker	52.62	59.83		45.93	
**Drinking (%)**			<0.0001		0.0212
No	22.48	34.00		26.81	
Yes	77.52	66.00		73.19	
**Sleep disturbances (%)**			0.1988		0.0001
No	75.86	73.21		66.95	
Yes	24.14	26.79		33.05	
**Diabetes (%)**			0.7142		<0.0001
No	91.00	91.37		84.14	
Yes	9.00	8.63		15.86	
**Mental health (%)**			<0.0001		<0.0001
Not depressed	93.94	88.62		85.04	
Depressed	6.06	11.38		14.96	
**BMI**	28.62 ± 6.46	27.70 ± 6.59	0.0021	30.15 ± 7.41	0.0008
**WC**	98.16 ± 16.02	94.74 ± 15.99	<0.0001	101.85 ± 17.67	<0.0001
**RFM**	34.50 ± 8.34	36.72 ± 7.90	<0.0001	37.31 ± 8.63	<0.0001
**WHtR**	0.58 ± 0.09	0.57 ± 0.09	0.0712	0.61 ± 0.10	<0.0001
**WWI**	10.85 ± 0.80	10.90 ± 0.83	0.0942	11.16 ± 0.84	<0.0001
**BRI**	5.15 ± 2.17	5.00 ± 2.21	0.1004	5.90 ± 2.51	<0.0001
**ABSI**	0.081 ± 0.005	0.081 ± 0.005	0.4785	0.082 ± 0.005	<0.0001

BMI, body mass index; WC, waist circumference; RFM, relative fat mass; WHtR, waist-to-height ratio; WWI, weight-adjusted waist index; BRI, body roundness index; ABSI, a body shape index. N is the number of participants; data are expressed as number (weighted percentage) or mean (standard deviation).

### 3.2 Association of seven obesity-related anthropometric indicators and chronic constipation

This study used weighted multivariable logistic regression to assess associations between chronic intestinal disorders and obesity-related indices, with constipation-related results detailed in Table 2. Analyzed as quartiles, WC, BMI, BRI, and WHtR consistently showed significant negative associations with constipation across three models. In contrast, WWI exhibited no statistically significant association with constipation in either categorical or continuous analyses (all *P* trend > 0.05). While ABSI exhibited no statistical significance in Model 1 (*P* trend = 0.452), subsequent adjustments for covariates revealed significant positive associations in both Models 2 (*P* trend < 0.01) and 3 (*P* trend < 0.05). For RFM, Model 1 revealed a significant positive association with constipation (*P* trend < 0.001), whereas Model 2 showed no significant association (*P* trend = 0.058), and Model 3 indicated a significant negative association (*P* trend < 0.01). When analyzed continuously, RFM maintained a positive association in Model 1 (*P* trend < 0.001) but shifted to significant negative associations in Models 2 (*P* trend < 0.05) and 3 (*P* trend < 0.01).

**Table 2:** Weighted logistic regression assessing the obesity-constipation relationship.

**Exposure**	**Model 1 [OR (95% CI)]**	**Model 2 [OR (95% CI)]**	**Model 3 [OR (95% CI)]**
**RFM** (Continuous)	1.033 (1.022, 1.044)^***^	0.983 (0.968, 0.999)^*^	0.974 (0.958, 0.990)^**^
Quartile 1 (≤29)	Reference	Reference	Reference
Quartile 2 (29–34.6)	1.311 (0.982, 1.751)	0.962 (0.727, 1.273)	0.954 (0.718, 1.269)
Quartile 3 (34.6–42.3)	2.052 (1.579, 2.667)^***^	0.890 (0.620, 1.276)	0.835 (0.580, 1.202)
Quartile 4 (>42.3)	2.010 (1.538, 2.627)^***^	0.729 (0.509, 1.045)	0.599 (0.409, 0.879)^*^
*P* for trend	<0.0001	0.058	<0.01
**WC**			
Quartile 1 (≤87.5)	Reference	Reference	Reference
Quartile 2 (87.5–97.7)	0.793 (0.602, 1.044)	0.914 (0.685, 1.221)	0.890 (0.666, 1.189)
Quartile 3 (97.7–108.3)	0.536 (0.422, 0.680)^***^	0.666 (0.510, 0.870)^**^	0.626 (0.479, 0.817)^**^
Quartile 4 (>108.3)	0.612 (0.489, 0.765)^***^	0.755 (0.594, 0.960)^*^	0.672 (0.520, 0.868)^**^
*P* for trend	<0.0001	<0.01	<0.001
**BMI**			
Quartile 1 (≤24.3)	Reference	Reference	Reference
Quartile 2 (24.3–27.9)	0.704 (0.539, 0.919)^*^	0.804 (0.610, 1.059)	0.787 (0.595, 1.041)
Quartile 3 (27.9–32.1)	0.752 (0.569, 0.995)^*^	0.870 (0.648, 1.169)	0.815 (0.604, 1.010)
Quartile 4 (>32.1)	0.595 (0.469, 0.755)^***^	0.594 (0.468, 0.754)^***^	0.530 (0.408, 0.689)^***^
*P* for trend	<0.001	<0.001	<0.0001
**WHtR**			
Quartile 1 (≤0.52)	Reference	Reference	Reference
Quartile 2 (0.52–0.58)	0.930 (0.750, 1.154)	0.997 (0.795, 1.251)	0.976 (0.782, 1.217)
Quartile 3 (0.58–0.65)	0.745 (0.588, 0.943)^*^	0.793 (0.610, 1.032)	0.726 (0.559, 0.942)^*^
Quartile 4 (>0.65)	0.814 (0.650, 1.019)	0.752 (0.597, 0.947)^*^	0.640 (0.497, 0.825)^**^
*P* for trend	<0.05	<0.01	<0.001
**WWI** (Continuous)	1.078 (0.988, 1.177)	1.040 (0.937, 1.155)	0.962 (0.863, 1.072)
Quartile 1 (≤10.4)	Reference	Reference	Reference
Quartile 2 (10.4–11)	0.966 (0.779, 1.198)	0.995 (0.803, 1.235)	0.965 (0.769, 1.210)
Quartile 3 (11–11.6)	1.067 (0.886, 1.284)	1.061 (0.850, 1.324)	0.973 (0.778, 1.217)
Quartile 4 (>11.6)	1.153 (0.946, 1.404)	1.073 (0.856, 1.346)	0.910 (0.708, 1.168)
*P* for trend	0.108	0.470	0.477
**BRI**			
Quartile 1 (≤3.78)	Reference	Reference	Reference
Quartile 2 (3.78–5.02)	0.930 (0.750, 1.154)	0.997 (0.795, 1.251)	0.976 (0.782, 1.217)
Quartile 3 (5.02–6.53)	0.745 (0.588, 0.943)^*^	0.793 (0.610, 1.032)	0.726 (0.559, 0.942)^*^
Quartile 4 (>6.53)	0.814 (0.650, 1.019)	0.752 (0.597, 0.947)^*^	0.640 (0.497, 0.825)^**^
*P* for trend	<0.05	<0.01	<0.001
**ABSI**			
Quartile 1 (≤0.078)	Reference	Reference	Reference
Quartile 2 (0.078–0.081)	0.903 (0.732, 1.114)	1.070 (0.867, 1.321)	1.054 (0.841, 1.320)
Quartile 3 (0.081–0.085)	0.845 (0.712, 1.003)	1.101 (0.902, 1.344)	1.082 (0.879, 1.331)
Quartile 4 (>0.085)	0.972 (0.822, 1.149)	1.359 (1.109, 1.667)^**^	1.262 (1.014, 1.571)^*^
*P* for trend	0.452	<0.01	<0.05

Model 1: crude; Model 2: adjusted for age, sex, race; Model 3: adjusted for age, sex, race, education level, economic status, smoking, drinking, sleep disturbances, depression, and diabetes. CI, confidence interval; OR, odds ratio.

*** *P* value < 0.001,

** *P* value < 0.01,

* *P* value < 0.05.

In Model 3, using the first quartile of obesity-related indicators as the reference, constipation was significantly negatively associated with higher quartiles of WC (Q3: OR = 0.63, 95% CI: 0.48, 0.82; Q4: OR = 0.67, 95% CI: 0.52, 0.87, *P* trend < 0.001), WHtR (Q3: OR = 0.73, 95% CI: 0.56, 0.94; Q4: OR = 0.64, 95% CI: 0.50, 0.83, *P* trend < 0.001), and BRI (Q3: OR = 0.73, 95% CI: 0.56, 0.94; Q4: OR = 0.64, 95% CI: 0.50, 0.83, *P* trend < 0.001). Similarly, BMI (Q4: OR = 0.53, 95% CI: 0.41, 0.69, *P* trend < 0.001) and RFM (Q4: OR = 0.60, 95% CI: 0.41, 0.88, *P* trend < 0.01) in the fourth quartile displayed an inverse association with constipation risk, while ABSI (Q4: OR = 1.26, 95% CI: 1.01, 1.57, *P* trend < 0.05) showed a positive association.

### 3.3 Association of seven obesity-related anthropometric indicators and chronic diarrhea

All indicators showed significant associations with diarrhea risk across the three models (Table 3). In Model 3, we observed significant positive associations in the fourth quartile for WWI (OR = 1.94, 95% CI: 1.52, 2.47, *P* trend < 0.001), WC (OR = 1.52, 95% CI: 1.19, 1.95, *P* trend < 0.01), BMI (OR = 1.45, 95% CI: 1.13, 1.85, *P* trend < 0.001), RFM (OR = 1.87, 95% CI: 1.25, 2.79, *P* trend < 0.01), BRI (OR = 1.69, 95% CI: 1.31, 2.17, *P* trend < 0.001), and WHtR (OR = 1.69, 95% CI: 1.31, 2.17, *P* trend < 0.001). Additionally, significant positive associations were found in the third quartile for WWI (OR = 1.52, 95% CI: 1.16, 1.99, *P* trend < 0.001). Furthermore, ABSI demonstrated a significant positive relationship with diarrhea in both the second and fourth quartiles (Q2: OR = 1.35, 95% CI: 1.04, 1.75; Q4: OR = 1.48, 95% CI: 1.15, 1.90, *P* trend < 0.05).

**Table 3:** Weighted logistic regression assessing the obesity-diarrhea relationship.

**Exposure**	**Model 1 [OR (95% CI)]**	**Model 2 [OR (95% CI)]**	**Model 3 [OR (95% CI)]**
**RFM** (Continuous)	1.041 (1.030, 1.052)^***^	1.047 (1.029, 1.065)^***^	1.036 (1.018, 1.055)^***^
Quartile 1 (≤29)	Reference	Reference	Reference
Quartile 2 (29–34.6)	1.439 (1.051, 1.970)^*^	1.353 (0.983, 1.863)	1.301 (0.928, 1.826)
Quartile 3 (34.6–42.4)	1.436 (1.064, 1.936)^*^	1.388 (0.976, 1.975)	1.256 (0.856, 1.839)
Quartile 4 (>42.4)	2.472 (1.892, 3.230)^***^	2.302 (1.552, 3.416)^***^	1.870 (1.254, 2.790)^**^
*P* for trend	<0.0001	<0.0001	<0.01
**WC**			
Quartile 1 (≤88.2)	Reference	Reference	Reference
Quartile 2 (88.2–98.2)	1.120 (0.928, 1.551)	1.181 (0.917, 1.521)	1.159 (0.901, 1.492)
Quartile 3 (98.2–108.9)	1.217 (0.948, 1.562)	1.215 (0.937, 1.575)	1.148 (0.892, 1.477)
Quartile 4 (>108.9)	1.737 (1.370, 2.202)^***^	1.744 (1.367, 2.224)^***^	1.521 (1.186, 1.952)^**^
*P* for trend	<0.0001	<0.0001	<0.01
**BMI**			
Quartile 1 (≤24.5)	Reference	Reference	Reference
Quartile 2 (24.5–28.1)	1.015 (0.758, 1.358)	0.997 (0.744, 1.337)	0.995 (0.739, 1.341)
Quartile 3 (28.1–32.4)	1.092 (0.863, 1.382)	1.045 (0.825, 1.323)	1.021 (0.795, 1.311)
Quartile 4 (>32.4)	1.663 (1.302, 2.123)^***^	1.579 (1.236, 2.015)^***^	1.445 (1.130, 1.849)^**^
*P* for trend	<0.0001	<0.0001	<0.001
**WHtR**			
Quartile 1 (≤0.53)	Reference	Reference	Reference
Quartile 2 (0.53–0.59)	1.101 (0.869, 1.394)	1.021 (0.800, 1.303)	1.007 (0.789, 1.286)
Quartile 3 (0.59–0.65)	1.304 (0.993, 1.713)	1.163 (0.880, 1.537)	1.095 (0.827, 1.450)
Quartile 4 (>0.65)	2.268 (1.794, 2.866)^***^	1.917 (1.499, 2.451)^***^	1.685 (1.307, 2.173)^***^
*P* for trend	<0.0001	<0.0001	<0.0001
**WWI** (Continuous)	1.585 (1.429, 1.758)^***^	1.469 (1.306, 1.653)^***^	1.338 (1.183, 1.512)^***^
Quartile 1 (≤10.4)	Reference	Reference	Reference
Quartile 2 (10.4–11)	1.446 (1.120, 1.866)^**^	1.378 (1.060, 1.792)^*^	1.311 (0.993, 1.730)
Quartile 3 (11–11.6)	1.886 (1.458, 2.439)^***^	1.693 (1.293, 2.219)^***^	1.518 (1.155, 1.995)^**^
Quartile 4 (>11.6)	2.798 (2.264, 3.458)^***^	2.358 (1.870, 2.974)^***^	1.937 (1.516, 2.474)^***^
*P* for trend	<0.0001	<0.0001	<0.0001
**BRI**			
Quartile 1 (≤3.83)	Reference	Reference	Reference
Quartile 2 (3.83–5.09)	1.101 (0.869, 1.394)	1.021 (0.800, 1.303)	1.007 (0.789, 1.286)
Quartile 3 (5.09–6.64)	1.304 (0.993, 1.713)	1.163 (0.880, 1.537)	1.095 (0.827, 1.450)
Quartile 4 (>6.64)	2.268 (1.794, 2.866)^***^	1.917 (1.499, 2.451)^***^	1.685 (1.307, 2.173)^***^
*P* for trend	<0.0001	<0.0001	<0.0001
**ABSI**			
Quartile 1 (≤0.078)	Reference	Reference	Reference
Quartile 2 (0.078–0.082)	1.417 (1.104, 1.819)^**^	1.435 (1.116, 1.847)^**^	1.345 (1.038, 1.744)^*^
Quartile 3 (0.082–0.085)	1.232 (0.963, 1.577)	1.230 (0.959, 1.578)	1.110 (0.859, 1.434)
Quartile 4 (>0.085)	1.886 (1.519, 2.343)^***^	1.758 (1.385, 2.231)^***^	1.475 (1.148, 1.895)^**^
*P* for trend	<0.0001	<0.001	<0.05

Model 1: crude; Model 2: adjusted for age, sex, race; Model 3: adjusted for age, sex, race, education level, economic status, smoking drinking, sleep disturbances, depression, and diabetes. CI, confidence interval; OR, odds ratio.

*** *P* value < 0.001,

** *P* value < 0.01,

* *P* value < 0.05.

### 3.4 Predictive value of seven indicators for chronic constipation and diarrhea

**Figure 2:**
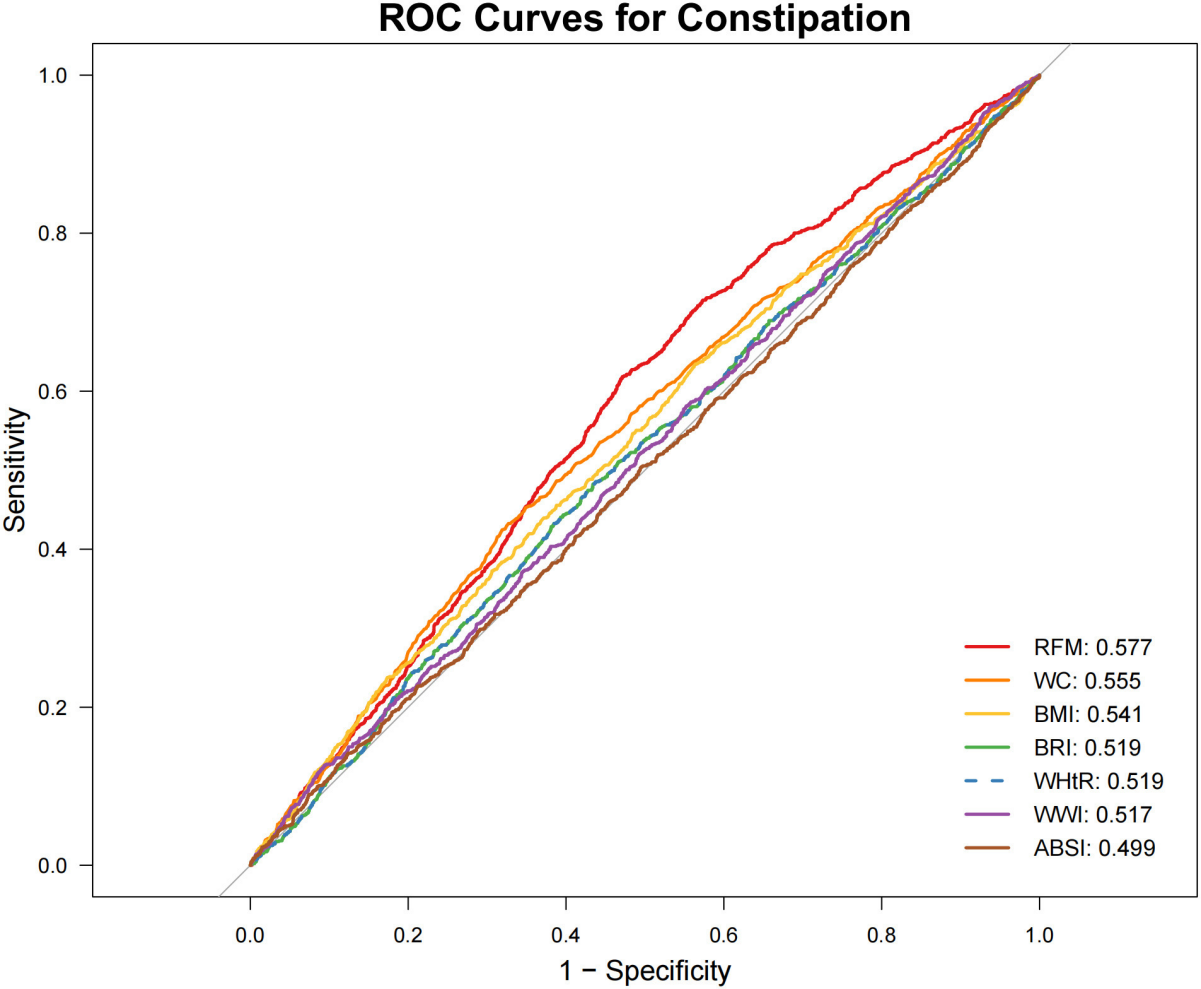
Receiver operating characteristic (ROC) curve evaluation of obesity-related indicators for predicting constipation. ABSI, a body shape index; BMI, body mass index; BRI, body roundness index; RFM, relative fat mass; WC, waist circumference; WHtR, waist-to-height ratio; WWI, weight-adjusted waist index.

**Figure 3:**
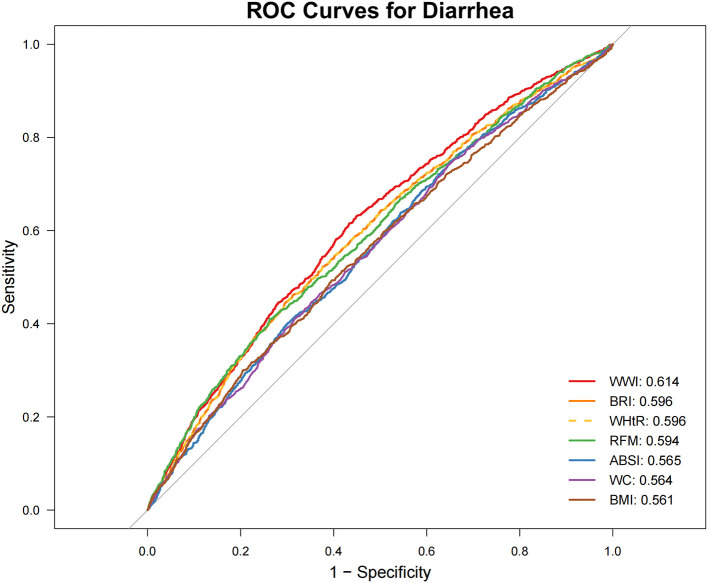
Receiver operating characteristic (ROC) curve evaluation of obesity-related indicators for predicting diarrhea. ABSI, a body shape index; BMI, body mass index; BRI, body roundness index; RFM, relative fat mass; WC, waist circumference; WHtR, waist-to-height ratio; WWI, weight-adjusted waist index.

ROC Analysis (Figures 2, 3; Table 4) identified RFM as the adiposity measure with the highest discriminative ability for constipation (AUC = 0.577), which significantly outperformed other adiposity measures (DeLong's test, all *P* < 0.05). For diarrhea, WWI exhibited the highest discriminative power (AUC = 0.614), while BRI and WHtR demonstrated similar accuracy (both AUC = 0.596). Statistical comparisons confirmed WWI's significantly better performance compared to alternative indices (DeLong's test, all *P* < 0.05).

**Table 4:** Results of ROC analysis of seven obesity-related indicators.

**Anthropometric measures**	**Best thresholds**	**Sensitivity**	**Specificity**	**AUC (95% CI)**	***P* for difference in AUC**
**Constipation**					
RFM	35.094	0.618	0.529	0.577 (0.559, 0.594)	Reference
WC	90.950	0.425	0.681	0.555 (0.536, 0.574)	<0.0001
BMI	28.915	0.634	0.436	0.541 (0.522, 0.560)	<0.0001
WHtR	0.568	0.483	0.566	0.519 (0.500, 0.538)	<0.0001
BRI	4.719	0.483	0.566	0.519 (0.500, 0.538)	<0.0001
WWI	12.068	0.127	0.906	0.517 (0.499, 0.536)	<0.0001
ABSI	0.087	0.142	0.876	0.499 (0.480, 0.518)	<0.0001
**Diarrhea**					
WWI	11.084	0.631	0.551	0.614 (0.595, 0.632)	Reference
BRI	6.199	0.446	0.704	0.596 (0.578, 0.615)	<0.01
WHtR	0.633	0.446	0.704	0.596 (0.578, 0.615)	<0.01
RFM	41.642	0.410	0.738	0.594 (0.575, 0.612)	0.019
ABSI	0.084	0.398	0.702	0.565 (0.546, 0.584)	<0.0001
WC	92.450	0.740	0.355	0.564 (0.545, 0.582)	<0.0001
BMI	29.515	0.493	0.604	0.561 (0.542, 0.580)	<0.0001

AUC, area under the curve; CI, confidence interval.

### 3.5 Exploring the exposure-risk relationship between the strongest predictor and bowel habits

#### 3.5.1 Non-linear association

**Figure 4:**
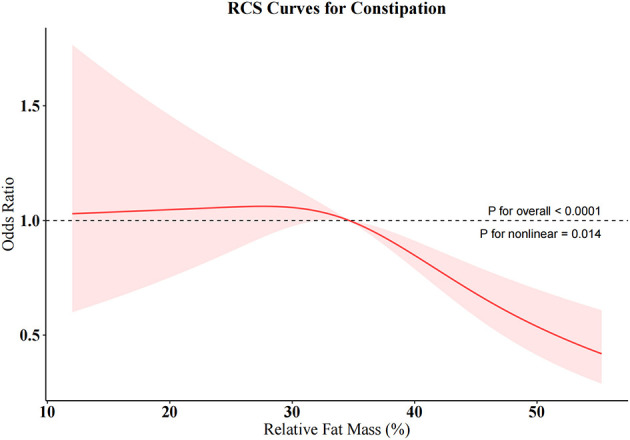
Restricted cubic spline (RCS) curve for RFM and constipation.

**Figure 5:**
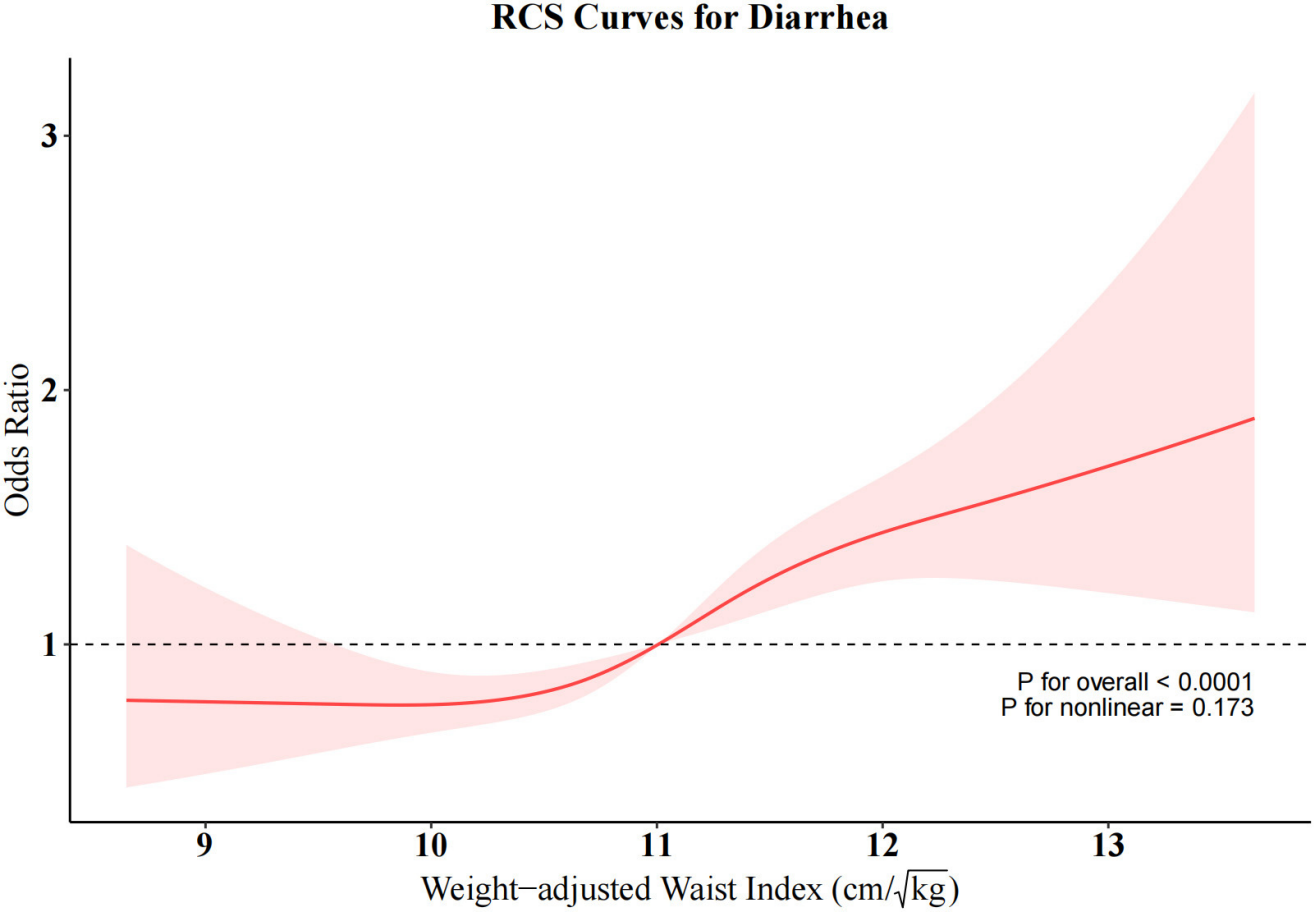
Restricted cubic spline (RCS) curve for WWI and diarrhea.

To further investigate the relationships between RFM and constipation as well as between WWI and diarrhea, we conducted weighted RCS analyses (Figures 4, 5). The restricted cubic spline regression with full covariate adjustment revealed a significant non-linear inverse association between RFM and constipation risk (*P* overall < 0.05, *P* non-linear = 0.014), while WWI exhibited a significant linear relationship with chronic diarrhea, showing a positive association with diarrhea risk (*P* overall < 0.05, *P* non-linear = 0.173).

#### 3.5.2 Subgroup analysis

Subgroup analyses stratified by gender, age, race, drinking/smoking status, sleep disturbances, psychological status, and diabetes mellitus were conducted to assess potential effect modification (Table 5). Interaction tests demonstrated no statistically significant moderating effects for any of these baseline characteristics (all interaction *P*-values > 0.05), confirming the robustness of both the RFM-constipation and WWI-diarrhea associations across all evaluated demographic and clinical subgroups.

**Table 5:** Subgroup analysis of RFM and constipation, alongside WWI and diarrhea.

**Variable**	**RFM and constipation**	***P* for interaction**	**WWI and diarrhea**	***P* for interaction**
**Sex**		0.215		0.952
Male	0.99 (0.97, 1.01)		1.04 (1.01, 1.07)	
Female	0.97 (0.95, 0.99)		1.04 (1.02, 1.06)	
**Age**		0.118		0.807
< 60	0.98 (0.97, 1.00)		1.04 (1.02, 1.06)	
≥60	0.93 (0.90, 0.96)		1.04 (1.01, 1.07)	
**Race**		0.099		0.420
Mexican American	0.99 (0.96, 1.02)		1.04 (1.00, 1.08)	
Non-Hispanic White	0.97 (0.95, 0.98)		1.03 (1.01, 1.06)	
Non-Hispanic Black	1.00 (0.97, 1.02)		1.05 (1.02, 1.08)	
Others	0.95 (0.91, 0.99)		1.05 (1.01, 1.09)	
**Smoking**		0.692		0.806
Never smoker	0.96 (0.95, 0.98)		1.05 (1.02, 1.08)	
Former smoker	0.98 (0.95, 1.02)		1.03 (1.00, 1.07)	
Current smoker	0.99 (0.96, 1.02)		1.03 (0.99, 1.07)	
**Drinking**		0.184		0.492
No	0.96 (0.94, 0.98)		1.05 (1.02, 1.09)	
Yes	0.98 (0.96, 1.00)		1.03 (1.01, 1.05)	
**Sleep disturbances**		0.545		0.490
No	0.97 (0.96, 0.99)		1.03 (1.01, 1.05)	
Yes	0.97 (0.95, 0.99)		1.06 (1.03, 1.09)	
**Diabetes**		0.508		0.338
No	0.97 (0.96, 0.99)		1.03 (1.02, 1.05)	
Yes	0.96 (0.90, 1.02)		1.07 (1.01, 1.14)	
**Mental health**		0.716		0.411
Not depressed	0.97 (0.95, 0.98)		1.04 (1.02, 1.06)	
Depressed	1.00 (0.96, 1.04)		1.03 (1.00, 1.06)	

#### 3.5.3 Sensitivity analysis

Sensitivity analyses, conducted after excluding participants with potential confounding conditions (pregnancy, colorectal cancer, or use of gastrointestinal/psychotropic/opioid medications) and adjusting for dietary and physical activity covariates, yielded robust associations (Table 6, Supplementary Figures 1, 2). The analyses confirmed a significant inverse association between RFM and constipation risk (*P* < 0.01) and a persistent positive association between WWI and diarrhea risk (*P* < 0.001). Among all obesity indicators, RFM exhibited the highest predictive ability for constipation (AUC = 0.579), while WWI showed the strongest predictive performance for diarrhea (AUC = 0.615).

**Table 6:** Sensitivity analysis of RFM and constipation, alongside WWI and diarrhea.

**WWI**	**Diarrhea**		**RFM**	**Constipation**	
	**OR (95% CI)**	***P*** **-value**		**OR (95% CI)**	***P*** **-value**
Continuous	1.427 (1.205, 1.689)	<0.001	Continuous	0.971(0.952, 0.990)	<0.01
**Classification**			**Classification**		
Quartile 1 (≤10.3)	reference		Quartile 1 (≤28.5)	reference	
Quartile 2 (10.3–10.9)	1.306 (0.934, 1.824)	0.111	Quartile 2 (28.5–33.5)	1.062 (0.693, 1.627)	0.771
Quartile 3 (10.9–11.4)	1.245 (0.911, 1.702)	0.158	Quartile 3 (33.5–41.0)	1.039 (0.664, 1.627)	0.859
Quartile 4 (>11.4)	2.269 (1.635, 3.148)	<0.001	Quartile 4 (>41.0)	0.555 (0.345, 0.894)	<0.01
P for trend		<0.001	P for trend		<0.01

Adjusted for age, sex, race, education level, economic status, smoking drinking, sleep disturbances, depression, diabetes, physical activity, daily total fat, dietary fiber, protein, carbohydrates, total sugar, caffeine, and energy intake levels.

CI, confidence interval; OR, odds ratio.

## 4 Discussion

This study contributes by comprehensively evaluating the relationship between seven anthropometric indices and bowel disorders and is the first to explore the association between two emerging indicators (RFM and ABSI) and chronic constipation and diarrhea. Using cross-sectional data from the 2005–2010 NHANES database and weighted multivariable logistic regression, we found that all obesity indicators, except WWI, were significantly associated with constipation, while all indicators were positively associated with diarrhea. ROC analysis identified RFM and WWI as the indicators with the highest discriminative power for predicting constipation and diarrhea, respectively. RCS analysis revealed a non-linear relationship between RFM and constipation risk, while a linear relationship was observed between WWI and diarrhea risk. Subgroup analyses demonstrated that the associations between RFM, WWI, and bowel disorders were stable and consistent across various confounding factors. Sensitivity analysis confirmed the robustness of the results. Notably, higher RFM was associated with a lower risk of constipation, while higher WWI was linked to a higher risk of diarrhea. ABSI showed a positive association with constipation, particularly in its highest quartile, where the risk of constipation was significantly elevated. These findings highlight RFM and WWI as clinically useful indicators for evaluating obesity's association with chronic constipation and diarrhea, respectively.

Despite its widespread use, BMI inadequately reflects adipose distribution patterns. Although computed tomography (CT) and magnetic resonance imaging (MRI) can precisely evaluate visceral fat distribution, their time-consuming procedures and high costs preclude routine clinical application. Novel indices including RFM, WWI, BRI, and ABSI demonstrate distinct advantages as accessible, cost-effective, and non-invasive measures for assessing body size and fat distribution. These indices show superior accuracy to BMI in evaluating both generalized and abdominal obesity, thereby enhancing their prognostic utility (19–22). The RFM provides an accurate estimation of total body fat percentage as validated by dual-energy X-ray absorptiometry (DXA) and demonstrates strong correlation with trunk adipose tissue levels. The proposed RFM cut-off values for obesity diagnosis are approximately 40% in women and 30% in men (25). The predictive capacity of RFM and WWI for cardiovascular diseases, depression, gallstones, diabetes mellitus, and other related conditions has been well-established (25–28). This study found that obesity was associated with both constipation and diarrhea, with varying degrees of obesity correlating to different bowel habits. While all obesity-related indicators showed significant positive associations with diarrhea, their relationship with constipation was complex and diverse. Fat distribution and obesity type may be important factors affecting constipation risk. Overall obesity indicators (BMI), absolute abdominal fat volume (WC), body fat distribution (BRI, WHtR), and body fat percentage (RFM) are negatively associated with constipation risk. Compared to individuals with normal weight, overweight/obese individuals exhibit characteristic gastrointestinal functional changes, including reduced satiety, adaptive gastric dilation, accelerated gastric emptying, and lower postprandial peak levels of serum PYY. These changes are particularly pronounced in individuals with abnormal waist circumference (29). A study of 354 constipation patients revealed that overweight patients had shorter rectosigmoid and total colonic transit times compared to those with normal BMI, along with improved colonic motility, stool consistency, and defecation frequency (30). In slow-transit constipation (STC) patients, a higher proportion had low BMI, which may be linked to reduced plasma motilin release (31). A meta-analysis of 14 observational studies demonstrated that overweight status in adults was inversely associated with constipation risk, whereas the opposite trend was observed in children (32). Low BMI and reduced abdominal fat were both associated with an increased risk of constipation and harder stools (15). Multiple studies suggest that underweight individuals are more prone to constipation, indicating that maintaining adequate visceral and subcutaneous fat levels is crucial for preventing chronic constipation (33, 34).

However, not all types of obesity or body composition states are negatively correlated with constipation. The WWI is calculated by normalizing WC to body weight. Some studies found that as WWI increases, fat mass rises while muscle and bone mass decrease, suggesting that WWI is associated with sarcopenic obesity (35). Similarly, other studies have shown that muscle mass is negatively correlated with ABSI, suggesting that ABSI may not only be a marker of visceral obesity but also an indicator of reduced muscle mass, potentially helping identify sarcopenia risk in overweight/obese individuals (36). The unique calculation method of ABSI may give it a distinct advantage in identifying the visceral fat-muscle ratio imbalance associated with constipation. Thus, we hypothesize that increased fat mass itself may not be the primary cause of constipation; rather, the quality and distribution of body composition may play a key role. Muscles, particularly core and pelvic floor muscles, are essential for generating adequate intra-abdominal pressure and coordinating defecation movements. Muscle loss may impair intra-abdominal pressure production and straining efficiency during defecation (37). A retrospective cross-sectional study found that constipation severity was positively correlated with sarcopenia in older adults. During defecation, muscle weakness caused by sarcopenia may lead to pelvic floor dysfunction and/or reduced abdominal pressure, contributing to defecatory disorders (38). Future research should incorporate direct measurements of muscle mass and visceral fat (e.g., bioelectrical impedance analysis, imaging) to validate the independent effects of obesity type and fat distribution characteristics on constipation risk.

Notably, clinical studies have shown that visceral adiposity leads to chronic constipation in Crohn's disease (CD) patients during clinical remission (39). Research has shown that individuals with obesity often display lower concentrations of growth hormone–releasing peptides while having higher levels of leptin. These changes in hormone levels might slow down gastric emptying and intestinal movement, possibly contributing to constipation development (40). Moreover, obesity may increase intestinal fat deposition, which disrupts hormone secretion and relaxes colonic smooth muscles, impairing intestinal motility and contributing to constipation (41, 42). Rectal hyposensitivity appears to be an important mechanistic factor in constipation development in the obese population. Additional studies are warranted to elucidate the mechanistic relationship between obesity/body fat distribution and gastrointestinal visceral sensitivity (43).

Obesity-associated gut dysbiosis facilitates pathogenic bacterial overgrowth, predisposing to diarrheal diseases such as ulcerative colitis and CD rather than constipation (44). Individuals with obesity often exhibit faster small intestinal motility and prolonged transit time in the distal colon, potentially resulting in impaired bile acid absorption and subsequent diarrhea (45, 46). A meta-analysis shows no significant correlation between constipation and obesity or elevated BMI, whereas diarrhea demonstrates a positive association with higher BMI levels. This association might stem from the increased osmotic load due to more food entering the small intestine, which in turn leads to enhanced intestinal secretion, faster stool transit to the colon, and softer stool consistency, ultimately causing diarrhea (16). Excessive adipose tissue, particularly visceral fat, induces adipokine dysregulation, chronic low-grade inflammation, and imbalances in gut microbiota, which collectively impair intestinal barrier function (manifested by increased permeability) and promote chronic diarrhea (47–49).

As a cross-sectional study, these findings demonstrate associations rather than causal relationships between obesity-related indices and constipation/diarrhea. The results suggest these indices may serve as practical and accessible biomarkers for identifying individuals with distinct bowel dysfunction patterns. Maintaining appropriate adiposity levels might help prevent chronic constipation while avoiding diarrhea risk. Prospective and clinical studies are warranted to verify the potential role of obesity-related indices in bowel disorder development and elucidate the underlying biological mechanisms.

This investigation offers notable strengths: (1) to the best of our knowledge, the first to evaluate seven adiposity metrics in relation to chronic bowel dysfunction, demonstrating that RFM and WWI exhibit the strongest associations with constipation and diarrhea respectively among all evaluated indices; (2) methodologically robust design incorporating population weighting, comprehensive covariate adjustment, and advanced analytics (weighted multivariable logistic regression, RCS, ROC, stratification). Limitations include inherent constraints of cross-sectional causality assessment, residual confounding from unmeasured or imprecisely measured variables despite extensive adjustments, and potential recall bias in self-reported symptom classification.

## 5 Conclusions

Seven obesity-related anthropometric indicators (WC, BMI, RFM, BRI, WWI, WHtR, ABSI) showed significant positive associations with diarrhea risk. Among these, WWI demonstrated the highest discriminative ability for diarrhea, displaying a linear relationship with the heightened risk of chronic diarrhea. In addition, all obesity indicators except WWI showed significant associations with constipation, with RFM demonstrating the strongest inverse association and a non-linear relationship with constipation risk. Although the obesity-diarrhea association is well-documented, elucidating the obesity-constipation relationship requires comprehensive consideration of multiple factors including age, sex, constipation subtype classification, adipose tissue distribution characteristics, and diverse obesity phenotypes. Future longitudinal studies are needed to clarify the temporal sequence and potential causal mechanisms underlying these associations, particularly through repeated anthropometric measurements and standardized bowel symptom assessments.

## Data Availability

The original contributions presented in the study are included in the article/Supplementary material, further inquiries can be directed to the corresponding author.

## Supplementary material

Supplementary Figure 1 ROC curves for constipation prediction in sensitivity analyses. ABSI, a body shape index; BMI, body mass index; BRI, body roundness index; RFM, relative fat mass; WC, waist circumference; WHtR, waist-to-height ratio; WWI, weight-adjusted waist index.

Supplementary Figure 2 ROC curves for diarrhea prediction in sensitivity analyses. ABSI, a body shape index; BMI, body mass index; BRI, body roundness index; RFM, relative fat mass; WC, waist circumference; WHtR, waist-to-height ratio; WWI, weight-adjusted waist index.

## References

[1] Kinoshita Y Ariyoshi R Fujigaki S Tanaka K Morikawa T Sanuki T. Endoscopic diagnosis of chronic diarrhea. DEN Open. (2022) 2:e53. 10.1002/deo2.53 35310743 PMC8828214

[2] Singh P Mitsuhashi S Ballou S Rangan V Sommers T Cheng V. Demographic and dietary associations of chronic diarrhea in a representative sample of adults in the United States. Am J Gastroenterol. (2018) 113:593–600. 10.1038/ajg.2018.24 29610515

[3] Bharucha AE Dunivan G Goode PS Lukacz ES Markland AD Matthews CA. Epidemiology, pathophysiology, and classification of fecal incontinence: state of the science summary for the National Institute of Diabetes and Digestive and Kidney Diseases (NIDDK) workshop. Am J Gastroenterol. (2015) 110:127–36. 10.1038/ajg.2014.396 25533002 PMC4418464

[4] Bharucha AE Lacy BE. Mechanisms, evaluation, and management of chronic constipation. Gastroenterology. (2020) 158:1232–49.e3. 10.1053/j.gastro.2019.12.034 31945360 PMC7573977

[5] Oh SJ Fuller G Patel D Khalil C Spalding W Nag A. Chronic constipation in the United States: results from a population-based survey assessing healthcare seeking and use of pharmacotherapy. Am J Gastroenterol. (2020) 115:895–905. 10.14309/ajg.0000000000000614 32324606 PMC7269025

[6] Bellini M Tonarelli S Barracca F Rettura F Pancetti A Ceccarelli L. Chronic constipation: is a nutritional approach reasonable? Nutrients. (2021) 13:3386. 10.3390/nu13103386 34684388 PMC8538724

[7] Zhao YF Guo XJ Zhang ZS Ma XQ Wang R Yan XY. Epidemiology of functional diarrhea and comparison with diarrhea-predominant irritable bowel syndrome: a population-based survey in China. PLoS ONE. (2012) 7:e43749. 10.1371/journal.pone.0043749 22937091 PMC3427143

[8] Afshin A Forouzanfar MH Reitsma MB Sur P Estep K Lee A. Health effects of overweight and obesity in 195 countries over 25 years. N Engl J Med. (2017) 377:13–27. 10.1056/NEJMoa1614362 28604169 PMC5477817

[9] Ward ZJ Bleich SN Cradock AL Barrett JL Giles CM Flax C. et al. Projected US state-level prevalence of adult obesity and severe obesity. N Engl J Med. (2019) 381:2440–50. 10.1056/NEJMsa1909301 31851800

[10] GBD 2021 US Obesity Forecasting Collaborators. National-level and state-level prevalence of overweight and obesity among children, adolescents, and adults in the USA, 1990–2021, and forecasts up to 2050. Lancet. (2024) 404:2278–98. 10.1016/S0140-6736(24)01548-4 39551059 PMC11694015

[11] Islam MR Arthur S Haynes J Butts MR Nepal N Sundaram U. The role of gut microbiota and metabolites in obesity-associated chronic gastrointestinal disorders. Nutrients. (2022) 14:624. 10.3390/nu14030624 35276983 PMC8838694

[12] Camilleri M Malhi H Acosta A. Gastrointestinal complications of obesity. Gastroenterology. (2017) 152:1656–70. 10.1053/j.gastro.2016.12.052 28192107 PMC5609829

[13] Moayyedi P. The epidemiology of obesity and gastrointestinal and other diseases: an overview. Dig Dis Sci. (2008) 53:2293–9. 10.1007/s10620-008-0410-z 18636328

[14] Silveira EA Santos A Ribeiro JN Noll M Dos Santos Rodrigues AP de Oliveira C. Prevalence of constipation in adults with obesity class II and III and associated factors. BMC Gastroenterol. (2021) 21:217. 10.1186/s12876-021-01806-5 33980157 PMC8114515

[15] Nagata N Sakamoto K Arai T Niikura R Shimbo T Shinozaki M. Effect of body mass index and intra-abdominal fat measured by computed tomography on the risk of bowel symptoms. PLoS ONE. (2015) 10:e0123993. 10.1371/journal.pone.0123993 25906052 PMC4408111

[16] Eslick GD. Gastrointestinal symptoms and obesity: a meta-analysis. Obes Rev. (2012) 13:469–79. 10.1111/j.1467-789X.2011.00969.x 22188520

[17] Eslick GD Talley NJ. Prevalence and relationship between gastrointestinal symptoms among individuals of different body mass index: a population-based study. Obes Res Clin Pract. (2016) 10:143–50. 10.1016/j.orcp.2015.05.018 26142872

[18] Klein S Allison DB Heymsfield SB Kelley DE Leibel RL Nonas C. Waist circumference and cardiometabolic risk: a consensus statement from shaping America's health: Association for weight management and obesity prevention; NAASO, the obesity society; the American society for nutrition; and the American diabetes association. Diabetes Care. (2007) 30:1647–52. 10.1038/oby.2007.632 17360974

[19] Woolcott OO Bergman RN. Relative fat mass (RFM) as a new estimator of whole-body fat percentage–a cross-sectional study in American adult individuals. Sci Rep. (2018) 8:10980. 10.1038/s41598-018-29362-1 30030479 PMC6054651

[20] Yang X Sun Z. Association between weight-adjusted-waist index and bowel habits. Sci Rep. (2024) 14:17658. 10.1038/s41598-024-66869-2 39085333 PMC11291746

[21] Thomas DM Bredlau C Bosy-Westphal A Mueller M Shen W Gallagher D. Relationships between body roundness with body fat and visceral adipose tissue emerging from a new geometrical model. Obesity (Silver Spring). (2013) 21:2264–71. 10.1002/oby.20408 23519954 PMC3692604

[22] Krakauer NY Krakauer JC. A new body shape index predicts mortality hazard independently of body mass index. PLoS ONE. (2012) 7:e39504. 10.1371/journal.pone.0039504 22815707 PMC3399847

[23] Patel CJ Pho N McDuffie M Easton-Marks J Kothari C Kohane IS. A database of human exposomes and phenomes from the US National Health and Nutrition Examination Survey. Sci Data. (2016) 3:160096. 10.1038/sdata.2016.96 27779619 PMC5079122

[24] Ballou S Katon J Singh P Rangan V Lee HN McMahon C. Chronic diarrhea and constipation are more common in depressed individuals. Clin Gastroenterol Hepatol. (2019) 17:2696–703. 10.1016/j.cgh.2019.03.046 30954714 PMC6776710

[25] Suthahar N Bergman RN de Boer RA. Replacing body mass index with relative fat mass to accurately estimate adiposity. Nat Rev Endocrinol. (2025) 21:393–4. 10.1038/s41574-025-01120-0 40312540 PMC13115555

[26] Zhu X Yue Y Li L Zhu L Cai Y Shu Y. The relationship between depression and relative fat mass (RFM): a population-based study. J Affect Disord. (2024) 356:323–8. 10.1016/j.jad.2024.04.031 38614443

[27] Wu X Song Y Wu S. The development and evaluation of nine obesity-based indices for gallstones in US adults. Int J Surg. (2025) 111:2348–57. 10.1097/JS9.0000000000002237 39869395 PMC12372735

[28] Shen Y Wu Y Luo P Fu M Zhu K Wang J. Association between weight-adjusted-waist index and depression in US adults: a cross-sectional study. J Affect Disord. (2024) 355:299–307. 10.1016/j.jad.2024.03.143 38548206

[29] Acosta A Camilleri M Shin A Vazquez-Roque MI Iturrino J Burton D. Quantitative gastrointestinal and psychological traits associated with obesity and response to weight-loss therapy. Gastroenterology. (2015) 148:537–546.e4. 10.1053/j.gastro.2014.11.020 25486131 PMC4339485

[30] Bouchoucha M Fysekidis M Rompteaux P Airinei G Sabate JM Benamouzig R. Influence of age and body mass index on total and segmental colonic transit times in constipated subjects. J Neurogastroenterol Motil. (2019) 25:258–66. 10.5056/jnm18167 30982242 PMC6474702

[31] Chen HB Huang Y Song HW Li XL He S Xie JT. Clinical research on the relation between body mass index, motilin and slow transit constipation. Gastroenterology Res. (2010) 3:19–24. 10.4021/gr2010.02.168w 27956980 PMC5139835

[32] Sun X Zhang S Zhou X. A causal association between obesity and constipation: a two-sample bidirectional Mendelian randomization study and meta-analysis. Front Nutr. (2024) 11:1430280. 10.3389/fnut.2024.1430280 39588045 PMC11586183

[33] Zhuang Y Li L Sun J Zhang Y Dai F. Association of body roundness index with chronic diarrhea and constipation, NHANES 2005-2010. J Health Popul Nutr. (2025) 44:50. 10.1186/s41043-025-00793-7 40022226 PMC11869572

[34] Yang X Wang M Ren L Shon K Cui G Cheng Y. Association between visceral adiposity index and bowel habits and inflammatory bowel disease: a cross-sectional study. Sci Rep. (2024) 14:23923. 10.1038/s41598-024-73864-0 39397029 PMC11471843

[35] Kim KJ Son S Kim KJ Kim SG Kim NH. Weight-adjusted waist as an integrated index for fat, muscle and bone health in adults. J Cachexia Sarcopenia Muscle. (2023) 14:2196–203. 10.1002/jcsm.13302 37550773 PMC10570086

[36] Biolo G Di Girolamo FG Breglia A Chiuc M Baglio V Vinci P. Inverse relationship between “a body shape index” (ABSI) and fat-free mass in women and men: insights into mechanisms of sarcopenic obesity. Clin Nutr. (2015) 34:323–7. 10.1016/j.clnu.2014.03.015 24814384

[37] Srinivasan SG Muthyala A Sharma M Feuerhak K Boon A Bailey KR. Abdomino-anal dyscoordination in defecatory disorders. Clin Gastroenterol Hepatol. (2022) 20:2091–101.e5. 10.1016/j.cgh.2021.11.040 34896282 PMC9174349

[38] Asaoka D Takeda T Inami Y Abe D Shimada Y Matsumoto K. Association between the severity of constipation and sarcopenia in elderly adults: a single-center university hospital-based, cross-sectional study. Biomed Rep. (2021) 14:2. 10.3892/br.2020.1378 33235719 PMC7678610

[39] Wan Y Zhang D Xing T Liu Q Chi Y Zhang H. The impact of visceral obesity on chronic constipation, inflammation, immune function and cognitive function in patients with inflammatory bowel disease. Aging. (2021) 13:6702–11. 10.18632/aging.202526 33675295 PMC7993735

[40] Wu T Rayner CK Young RL Horowitz M. Gut motility and enteroendocrine secretion. Curr Opin Pharmacol. (2013) 13:928–34. 10.1016/j.coph.2013.09.002 24060702

[41] Halim MA Degerblad M Sundbom M Karlbom U Holst JJ Webb DL. Glucagon-like peptide-1 inhibits prandial gastrointestinal motility through myenteric neuronal mechanisms in humans. J Clin Endocrinol Metab. (2018) 103:575–85. 10.1210/jc.2017-02006 29177486

[42] Wang L Gourcerol G Yuan PQ Wu SV Million M Larauche M. Peripheral peptide YY inhibits propulsive colonic motor function through Y2 receptor in conscious mice. Am J Physiol Gastrointest Liver Physiol. (2010) 298:G45–56. 10.1152/ajpgi.00349.2009 19892938 PMC2806102

[43] Lodhia NA Hiramoto B Horton L Goldin AH Thompson CC Chan WW. Obesity is associated with altered rectal sensitivity in chronic constipation. Dig Dis Sci. (2024) 69:884–91. 10.1007/s10620-023-08246-z 38184499 PMC10961196

[44] Jung SE Joo NS Han KS Kim KN. Obesity is inversely related to hydrogen-producing small intestinal bacterial overgrowth in non-constipation irritable bowel syndrome. J Korean Med Sci. (2017) 32:948–53. 10.3346/jkms.2017.32.6.948 28480652 PMC5426230

[45] Delgado-Aros S Camilleri M Garcia MA Burton D Busciglio I. High body mass alters colonic sensory-motor function and transit in humans. Am J Physiol Gastrointest Liver Physiol. (2008) 295:G382–8. 10.1152/ajpgi.90286.2008 18617555 PMC2519862

[46] Yang D Lyu C He K Pang K Guo Z Wu D. Bile acid diarrhea: from molecular mechanisms to clinical diagnosis and treatment in the era of precision medicine. Int J Mol Sci. (2024) 25:1544. 10.3390/ijms25031544 38338820 PMC10855108

[47] Turpin T Thouvenot K Gonthier MP. Adipokines and bacterial metabolites: a pivotal molecular bridge linking obesity and gut microbiota dysbiosis to target. Biomolecules. (2023) 13:1692. 10.3390/biom13121692 38136564 PMC10742113

[48] Gummesson A Carlsson LM Storlien LH Bäckhed F Lundin P Löfgren L. Intestinal permeability is associated with visceral adiposity in healthy women. Obesity (Silver Spring). (2011) 19:2280–2. 10.1038/oby.2011.251 21852815

[49] Lee CG Lee JK Kang YS Shin S Kim JH Lim YJ. Visceral abdominal obesity is associated with an increased risk of irritable bowel syndrome. Am J Gastroenterol. (2015) 110:310–9. 10.1038/ajg.2014.422 25583325

