# Supplementary figures and images for: Association of obesity-related anthropometric indicators with chronic constipation and diarrhea among U.S. adults: a cross-sectional study
Source: Front Nutr. 2025 Aug 18;12:1610214. doi: 10.3389/fnut.2025.1610214 (PMC12400860; doi:10.3389/fnut.2025.1610214)

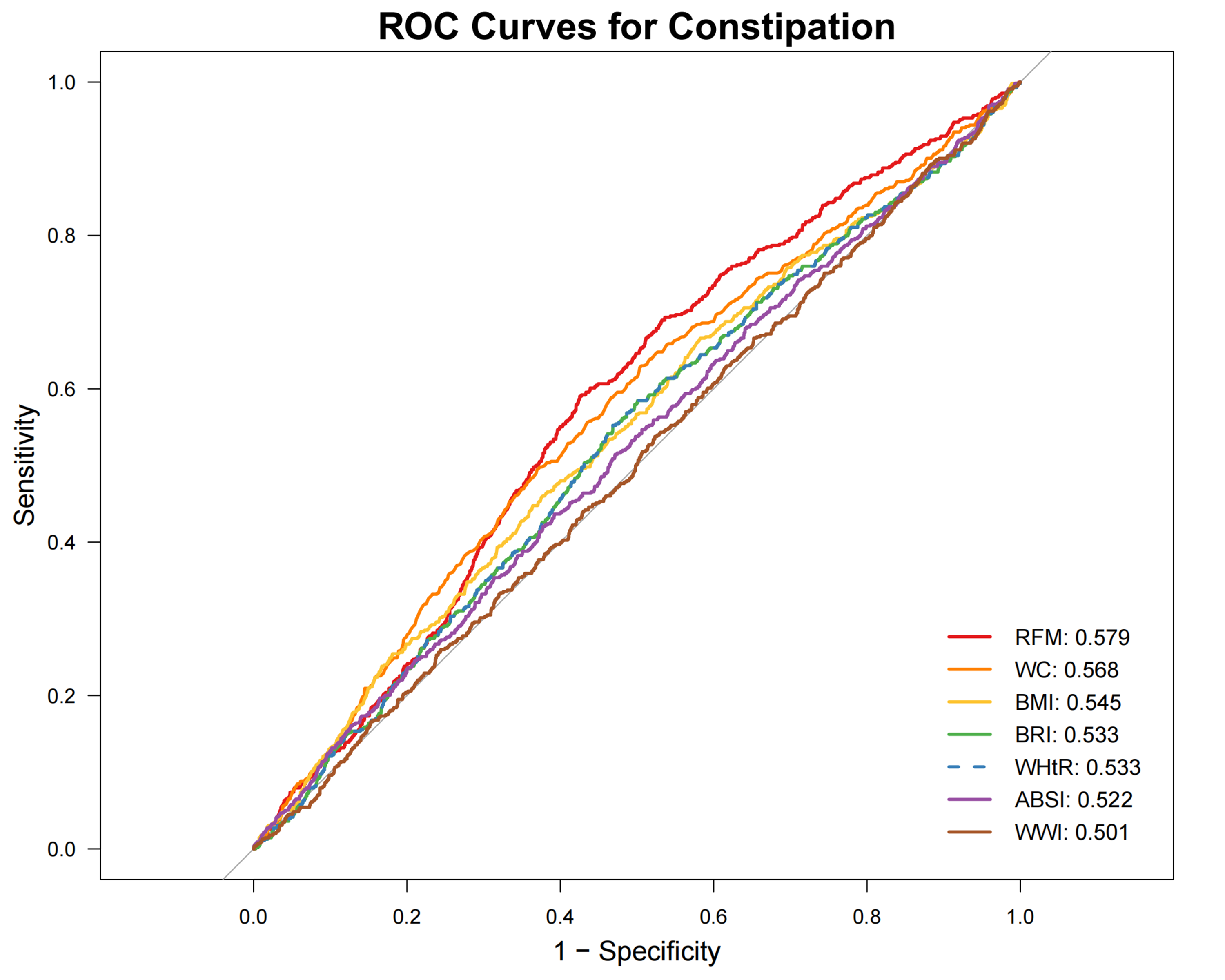

Supplement: Supplementary Figure 1 — ROC curves for constipation prediction in sensitivity analyses. ABSI, a body shape index; BMI, body mass index; BRI, body roundness index; RFM, relative fat mass; WC, waist circumference; WHtR, waist-to-height ratio; WWI, weight-adjusted waist index. [file Image_1.tif]

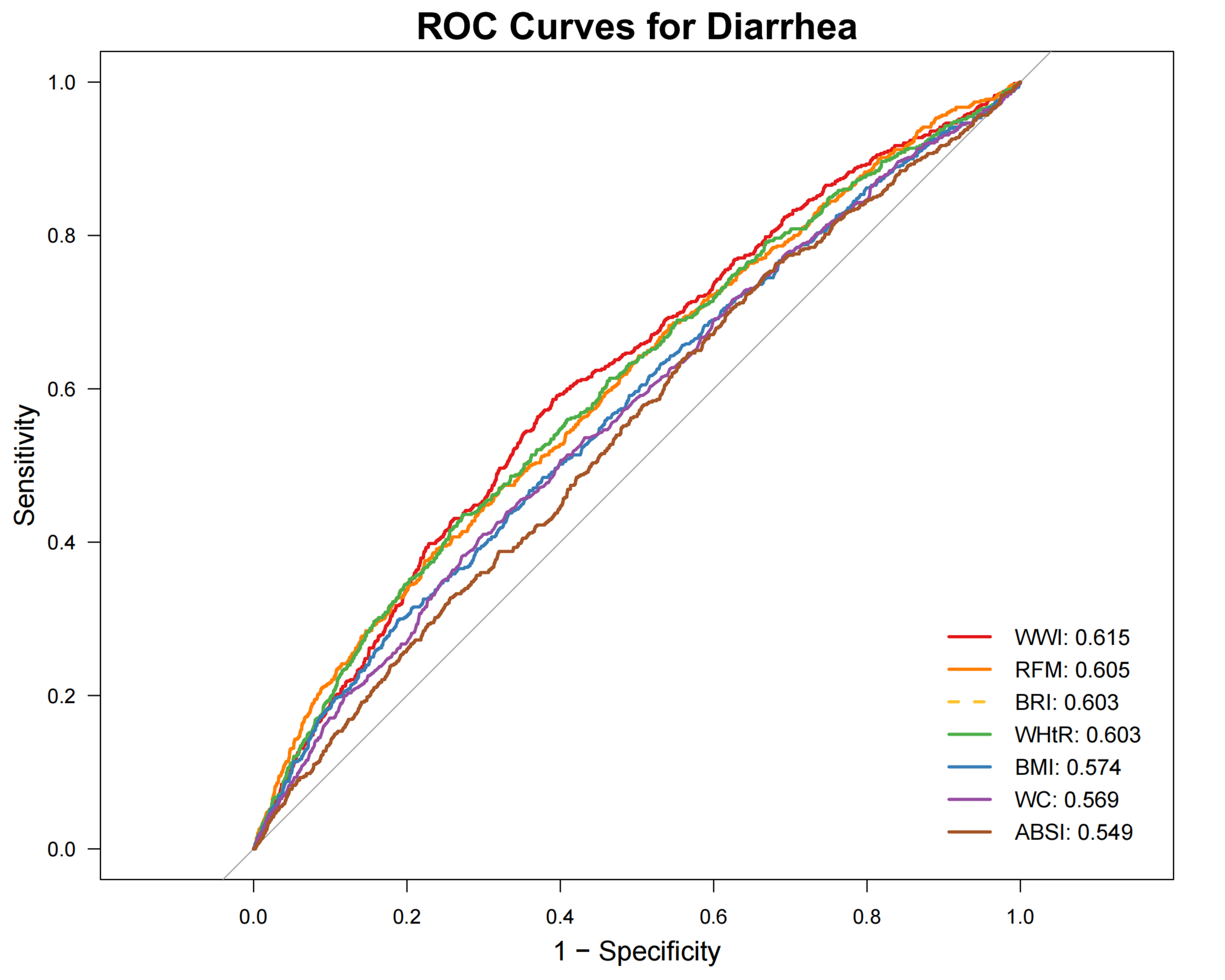

Supplement: Supplementary Figure 2 — ROC curves for diarrhea prediction in sensitivity analyses. ABSI, a body shape index; BMI, body mass index; BRI, body roundness index; RFM, relative fat mass; WC, waist circumference; WHtR, waist-to-height ratio; WWI, weight-adjusted waist index. [file Image_2.tif]
